# Relationship between Pollen Counts and Weather Variables in East-Mediterranean Coast of Turkey

**DOI:** 10.1080/10446670410001670544

**Published:** 2004-03

**Authors:** Derya Ufuk Altintaş, Gülbin Bingöl Karakoç, Mustafa Yilmaz, Münevver Pinar, Seval Güneçer Kendirli, Halil Çakan

**Affiliations:** 1 Faculty of Medicine Department of Pediatric Allergy and Immunology Çukurova University Turkey; 2 Department of Biology Faculty of Science University of Ankara Turkey; 3 Faculty of Biology University of Ankara Turkey

## Abstract

*Background*: Aeroallergen sampling provides information regarding the onset, duration and severity of the pollen season that clinicians use to guide allergen selection for skin testing and treatment.

*Objectives*: This atmospheric survey reports (1) airborne pollen contributions in Adana in one-year period (2) pollen onset, duration and peak level (3) the relationship between airborne pollen and selected meteorological variables and; (4) effects on symptoms in pollen allergic children.

*Methods*: Pollen sampling was performed with a volumetric Burkard Spore Trap. Meteorological data were measured daily from April 2001 to April 2002. Asthma symptom scores were investigated in 186 pollen allergic children that were on follow up in pediatric allergy outpatient clinics during same period.

*Results*: Average measurements included 82.5% tree pollen, 7.7% grass pollen and 9.8% herb pollen 54 taxa were identified during one year. The most prominent tree pollens were Cupressaceae, Eucalyptus and Pinus. The most common herb was Chenopodiaceae pollen family. When airborne pollen levels were examined in relation to single meteorological conditions; daily variations in total pollen counts were not significantly correlated with any variable studied (humidity, rainfall, temperature and wind) (*p*>0.05). On the other hand, statistically significant relationship between pollen concentration and symptom scores were found (*p*>0.05). Positive correlations were seen between both Gramineae and Herb pollen, and humidity and rainfall from March to July. However, positive correlations were detected between tree pollen counts and temperature and humidity in May and June.

*Conclusion*: This survey is the first volumetric airborne pollen analysis conducted in the survey area in Adana. This study suggested that the effects of weather on pollen count and symptom scores in this population could not be clearly identified with the evaluation of one-year data. However, pollen counts had effect on allergic symptoms in pollen allergic children. Examination of the complex interaction of multiple whether parameters would perhaps more fully elucidate the relationship between meteorology and aerobiology and provide the clinician with information necessary to forecast pollen prevalence. An awareness of the ever chancing, local aeroallergen patterns requires regular monitoring. Such awareness serves as a useful guide in the effective testing and treatment of atopic patients.


*Clinical & Developmental Immunology*, March 2004, Vol. 11 (1), pp. 87–96

Taylor & Francis healthsciences

### Relationship between Pollen Counts and Weather Variables in East-Mediterranean Coast of Turkey

### Does it Affect Allergic Symptoms in Pollen Allergic Children?

DERYA UFUK ALTINTAŞ[a,*], GÜLBIN BINGÖL KARAKOÇ[a], MUSTAFA YILMAZ[a], MÜNEVVER PINAR[b], SEVAL GÜNEŞER KENDIRLI[a] and HALIL ÇAKAN[c]

[a]*Faculty of Medicine, Department of Pediatric Allergy and Immunology, Çukurova University, Turkey;* [b]*Department of Biology, Faculty of Science, University of Ankara, Turkey;* [c]*Faculty of Biology, University of Ankara, Turkey*

*Background:* Aeroallergen sampling provides information regarding the onset, duration and severity of the pollen season that clinicians use to guide allergen selection for skin testing and treatment.

*Objectives:* This atmospheric survey reports (1) airborne pollen contributions in Adana in one-year period (2) pollen onset, duration and peak level (3) the relationship between airborne pollen and selected meteorological variables and; (4) effects on symptoms in pollen allergic children.

*Methods:* Pollen sampling was performed with a volumetric Burkard Spore Trap. Meteorological data were measured daily from April 2001 to April 2002. Asthma symptom scores were investigated in 186 pollen allergic children that were on follow up in pediatric allergy outpatient clinics during same period.

*Results:* Average measurements included 82.5% tree pollen, 7.7% grass pollen and 9.8% herb pollen 54 taxa were identified during one year. The most prominent tree pollens were *Cupressaceae*, *Eucalyptus* and *Pinus*. The most common herb was *Chenopodiaceae* pollen family. When airborne pollen levels were examined in relation to single meteorological conditions; daily variations in total pollen counts were not significantly correlated with any variable studied (humidity, rainfall, temperature and wind) ($p > 0.05$). On the other hand, statistically significant relationship between pollen concentration and symptom scores were found ($p > 0.05$). Positive correlations were seen between both Gramineae and Herb pollen, and humidity and rainfall from March to July. However, positive correlations were detected between tree pollen counts and temperature and humidity in May and June.

*Conclusion:* This survey is the first volumetric airborne pollen analysis conducted in the survey area in Adana. This study suggested that the effects of weather on pollen count and symptom scores in this population could not be clearly identified with the evaluation of one-year data. However, pollen counts had effect on allergic symptoms in pollen allergic children. Examination of the complex interaction of multiple whether parameters would perhaps more fully elucidate the relationship between meteorology and aerobiology and provide the clinician with information necessary to forecast pollen prevalence. An awareness of the ever chancing, local aeroallergen patterns requires regular monitoring. Such awareness serves as a useful guide in the effective testing and treatment of atopic patients.

### INTRODUCTION

Pollen-induced allergic diseases such as asthma, rhinitis and atopic dermatitis are important health problems all over the world. There is a body of evidence suggesting that prevalence of allergic diseases induced by pollens is on the increase in developed countries, a trend that is clearly evident also in the Mediterranean area (D'amato *et al.*, 1991, 1998; ERCHS, 1996; ISAAC, 1998). Avoiding exposure to allergens constitutes the first and also the most important phases in the treatment of allergic diseases. Clinicians use this information to identify appropriate allergens for skin testing and to guide pharmacologic and immunotherapeutic intervention and allergen avoidance. Thus, the determination of pollen map in an area is of great importance, since the study of pollinosis can not be limited to national boundaries.

Because of its mild winters and sunny days with dry summers, which are characteristic climatic conditions, the Mediterranean area is different from those of central and northern Europe. Classical examples of allergenic-pollen producing plants, which are typical of the Mediterranean climate, are *Parietaria*, *Olea* and *Cupressaceae* (Ozkaragöz, 1967; Bousquet *et al.*, 1985; D'amato *et al.*, 1991; Dvorin *et al.*, 2001).

*Corresponding author. E-mail: deryader@future.net

ISSN 1740-2522 print/ISSN 1740-2530 online © 2004 Taylor & Francis Ltd
DOI: 10.1080/10446670410001670544

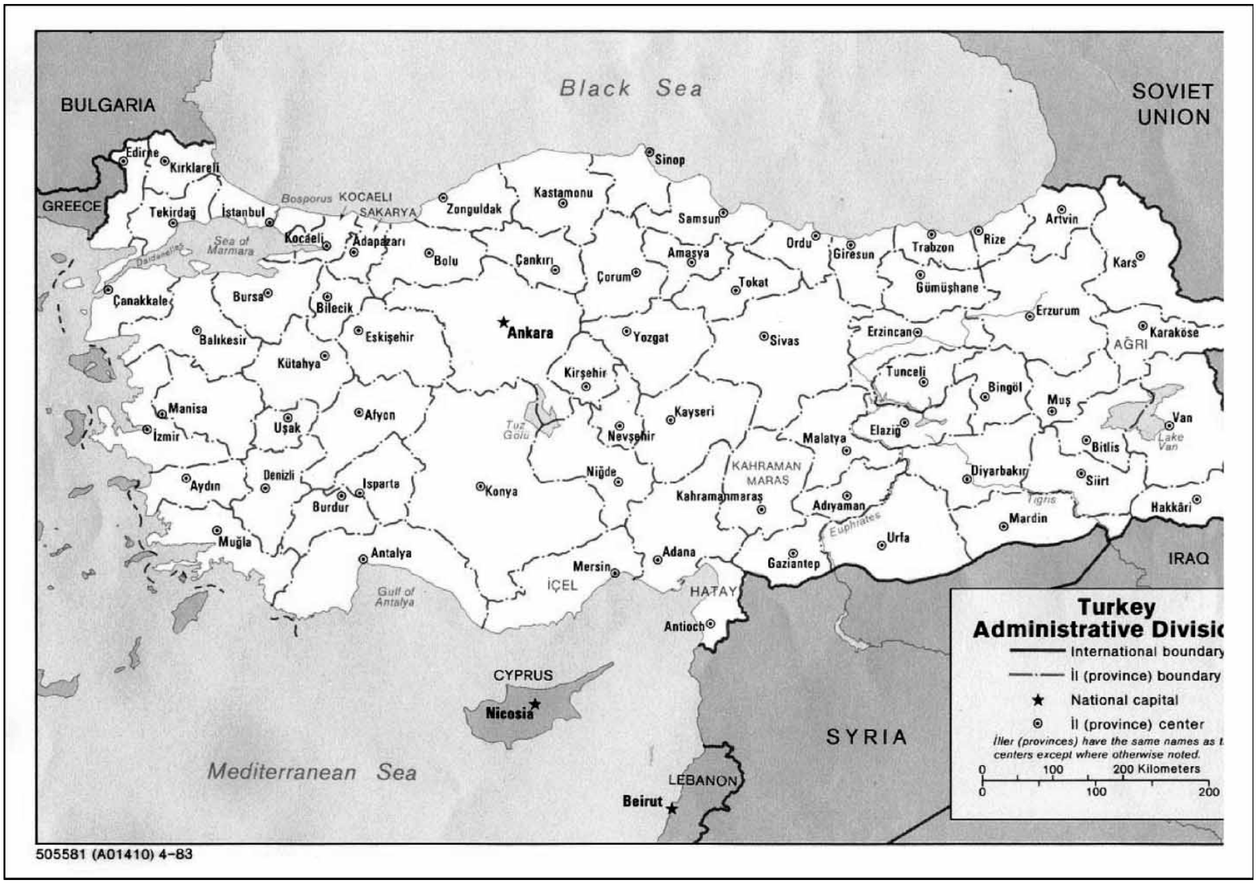

FIGURE 1 Turkey Map.

In the last 30 years or so, aerobiological studies have been developed rapidly in most parts of Europe and also in Mediterranean. In our region, pollen map and its changes in accordance with the climatic changes have not been studied before. In this study, (1) we investigated the types and counts of pollens and their changes according to months, (2) examined the relationship between pollen prevalence and selected meteorological variables and (3) demonstrated the effect on allergic symptoms in childhood.

Previously, a pollen calendar of Ankara for 10 years which is situated in North-east central Anatolia (Fig. 1) at an altitude of 820 m above sea level was made out for the first time (İnceoğlu *et al.*, 1994; Pınar *et al.*, 1999).

### SUBJECT AND METHODS

### Pollen Data

The pollens were collected weekly with Burkard® volumetric 7-day recording spore trap placed in the center of Adana from 1 April 2001 to 31 March 2002 (Fig. 1). The pollen grains and counts were investigated and calculated by the experienced aerobiolog. The tape was attached to a rotating drum which moved at 2 mm/h, completing a single revolution every seven days. At the end of the every 7-day cycle the tape was removed, attached to glass slides stained trypan blue and examined microscopically. The characteristics of pollen were defined using $\times 100$ immersion and $\times 10$ ocular, pollen count was made by $\times 40$ objective.

### Subject

Subjects were selected in pollen sensitive patients that applied for regular controls at Çukurova University Pediatric Allergy and Clinic Immunology out patient clinic during study period. There were 77 girls (41.4%) and 109 boys (58.6%) with the mean age of 11.42 ± 5.04 (min. 1.5/max. 18). All patients giving clinical history of allergic rhinitis and/or asthma related with pollen season were evaluated. The patients included to this study had skin prick test for pollen or serum pollen specific IgE. Distribution of the patients according to diagnosis was shown in Table I.

TABLE I The diagnosis of pollen allergic patients

| Diagnosis | N | % |
|---|---|---|
| Allergic Rhinitis | 94 | 50.5 |
| Asthma Bronchiole | 51 | 27.5 |
| Allergic Rhinitis + Asthma Bronchiole | 36 | 19.3 |
| The others | 5 | 2.7 |
| Total | 186 | 100 |

TABLE II Symptom scores

| | | | |
|---|---|---|---|
| Rhinitis medication | | Rhinitis symptom | |
| No medication | 0 | No symptom | 0 |
| Antihistamine/chromon | 1 | Mild | 1 |
| Nasal steroid and chromon | 2 | Moderate | 2 |
| Antihistamine and steroid | 3 | Severe | 3 |
| Oral or parenteral steroid | 4 | | |
| Asthma medication | | Asthma symptom | |
| No medication | 0 | No symptom | 0 |
| Inhale salbutamol 200 μ/wk | 1 | Mild | 1 |
| Inhaled salbutamol 600 μ/wk | 2 | Moderate | 2 |
| Chromons7leukotrien antagonists/ketotifen | 3 | Severe/depressed daily activity | 3 |
| Inhaled steroid | 4 | | |
| Oral aminocardol | 5 | | |
| Oral steroid < 20 mg | 6 | | |
| Oral steroid > 20 mg | 7 | | |

Common grass, weed and Mediterranean tree pollen specific IgE (Pharmacia CAP System, Sweden) were measured in all patients. Skin prick test was performed on the forearm of each subject by using major allergens (Allergopharma, Germany). Herb mixture, tree mixture, graminea, eucalyptus were used as allergens. Saline was used as negative control and histamine as positive control in all patients. Skin tests were defined as positive if the wheal size was more than 3 mm or equal to the wheal of positive control after 15 min.

Symptom scores (SS) (Table II) described by Bousquet *et al.* (1985) was used with a small modification. Symptom scores of all the patients were performed at every monthly visit.

### Weather Recordings

Meteorological data were obtained from the monthly bulletin of meteorological station in Adana center at an altitude of 70 m above sea level, located from the sampling site. A total of four parameters were selected for this investigation, temperature, relative humidity, wind and rainfall.

### Statistical Analysis

The softwares used for the data handling and analysis were SPSS for windows rel 10.0. All data were defined as mean ± SD. Spearmen correlation test and Cochran test used to investigate the relation between pollen count, meteorological variables and symptom scores. A significance level of 0.05 was adopted to be critical in all statistical calculations.

### RESULTS

Adana which has 14,030 km$^2$ surface area is located on east Mediterranean coast, in South-east Anatolia (Fig. 1). The city has a population of 10,50,000. It is surrounded by the Taurus Mountains covered with pine forest as vegetation specific to this area. It is also possible to see many of the steppe species in the abandoned areas, parks, gardens and roadsides in the city.

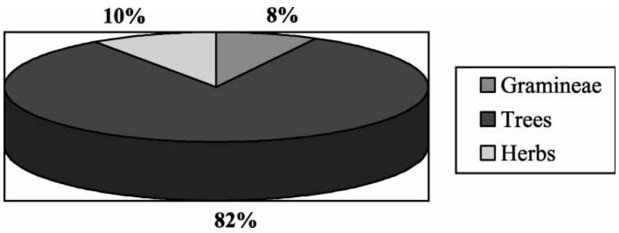

GRAPHIC 1 The distribution of pollens.

During observation of the one year, 54 taxa were determined in the atmosphere of Adana. Twenty nine of taxa belong to Gramineae. Plants with high pollen counts were classified into three groups as Gramineae, herb and trees. In Adana, total pollen count (in 1 m$^3$) is 32,694 pollen/m$^3$. Of the total pollen count in a year, 26,979 belonged to tree pollens, 3196 to herb and 2519 to Gramineae. Of the yearly total pollen count, three pollens compromised 82.5%, grass 9.8% and Gramineae 7.7% (Graphic 1, Table III).

Pollens were seen in all months, even in January. High levels of pollens were observed from the beginning of March till the end of June. During the summer, although temperature was on rise, pollen counts decreased. We assumed that this decrease in pollen counts was not a matter of the meteorological factors but it could be due to the termination of pollination period of trees, which can give a lot of pollen. Of the total pollens 82.5% were tree pollens that were found in parks and gardens, especially from March to June.

Two peaks of pollen counts were evident throughout the year, one in February, and another in May. The first peak was assumed because the beginning of pollinisation season of *Cupressaceae/Taxaceae* family. The second peak was due to high levels of *Eucalyptus*, *Pineaceae*, *Gramineae*, *Compositeae* taxa pollen counts. *Cupressaceae/Taxaceae*, *Eucalyptus*, *Morus*, *Oleaceae*, *Pineaceae*, *Quersus*, *Rosaceae* and *Salix* in tree pollens and predominantly Gramineae and then *Artemisia*, *Chenopodiaceae/Amaranthaceae*, *Compositea*, *Leguminosae*, *Plantago* and *Umbelliferae* in weeds were major pollens. Pollen counts were started to decrease in late June and reached to minimum levels in December.

### Tree Pollens

Tree pollens were the major components of the total pollens. *Cuppressacea / Taxaceae* was the most prevalent tree pollen. *Pinaceae* ,as another pollen producing tree was the most important and long durable in Adana. Adana has also got a lot of *Eucalyptus* trees which are very pollen producing tree as well. Generally, tree pollens started to

TABLE III The pollen concentration according to months in Adana (Pollen/m$^3$)

| | April 2001 | May 2001 | June 2001 | July 2001 | August 2001 | September 2001 | October 2001 | November 2001 | December 2001 | January 2002 | February 2002 | March 2002 | Total Pollen/m$^3$ |
|---|---|---|---|---|---|---|---|---|---|---|---|---|---|
| *Gramineae* (Poaceae) | 304 | 1223 | 403 | 95 | 133 | 122 | 35 | 20 | 6 | 2 | 2 | 174 | 2519 |
| *Herbs* | | | | | | | | | | | | | |
| Ambrosia | | | | | 27 | 31 | | | | | | | 58 |
| Artemisia | 4 | 7 | | | 73 | 80 | 31 | 4 | | | | | 199 |
| Carex | | 13 | 10 | | | 4 | | | | | | | 27 |
| Caryophyllaceae | 4 | 4 | | | | | | | | | | | 8 |
| Chenop/ Amaranth. | 7 | 32 | 78 | 31 | 379 | 452 | 44 | 8 | | 2 | | 4 | 1039 |
| Cistus | 4 | 7 | | | | | | | | | | | 11 |
| Compositae | 43 | 121 | 27 | 7 | 42 | 56 | 14 | 4 | | | | 4 | 318 |
| Cornus | | | 5 | | | | | | | | | | 5 |
| Crocus | | | | | | | | | 6 | | | | 6 |
| Cruciferae | 4 | 66 | 4 | | | | | | | | | | 74 |
| Ericaceae | 92 | 4 | 5 | | | | | | 2 | | | | 103 |
| Juncus | | 7 | | | | | | | | | | | 7 |
| Labiatae | 4 | 10 | 4 | 4 | | | 5 | | | | | | 27 |
| Leguminasae | 199 | 157 | 26 | 12 | 8 | | | 4 | | | 2 | 57 | 465 |
| Liliaceae | | 4 | 15 | | | | | | | | | | 19 |
| Linum | | 4 | | | | | | | | | | | 4 |
| Orchidaceae | 4 | | | | | | | | | | | | 4 |
| Papaver | | | | | | | | | | | | 5 | 5 |
| Plantago | 51 | 61 | 24 | | | | | | | | | 4 | 140 |
| Ranunculus | | 5 | | | | | | | | | | | 5 |
| Rhamnaceae | 7 | 17 | | | | | | | | | | | 24 |
| Rubiaceae | 13 | 10 | 7 | | | | | | | | | | 30 |
| Rumex | 24 | 48 | 12 | | | | | | | 12 | 2 | 4 | 106 |
| Typha | | 12 | | | | | | | | | | | 12 |
| Umbelliferae | 162 | 92 | 102 | 4 | 48 | | | | | | | | 408 |
| Gentianaceae | 4 | | | | | | | | | | | | 4 |
| Scrophulariaceae | 15 | | | | | | | | | | | | 15 |
| Urticaceae | 22 | 16 | 6 | | | | | | 2 | 4 | | 25 | 73 |
| *Trees* | | | | | | | | | | | | | |
| Acer | 90 | 133 | 4 | | | | | | | | | 32 | 259 |
| Ailanthus | | 39 | | | | | | | | | | | 39 |
| Betula | 205 | 85 | | | | 5 | | 626 | 114 | 4 | | 6 | 1045 |
| Carpinus | 32 | 38 | | | | | | | 17 | | 2 | | 89 |
| Cupres/ Taxaceae | 221 | 916 | 56 | | 17 | | | 4 | 16 | 275 | 15242 | 912 | 17659 |
| Ericaceae | | | | | | | | | 5 | 2 | | | 7 |
| Eucalyptus | 153 | 1369 | 78 | | | | 8 | 13 | 8 | | | | 1629 |
| Fagus | 17 | 30 | | | | | | | | | | | 47 |
| Fraxinus | 7 | 4 | | | | | | | | 3 | 12 | 12 | 38 |
| Juglans | 7 | 4 | | | | | | | 2 | | | 16 | 29 |
| Liquidambar | | | | | | | | | | | | 4 | 4 |
| Morus | 143 | 24 | 7 | | | | | | 2 | | | 636 | 812 |
| Oleaceae | 189 | 211 | 15 | | | | | | | | | 2 | 414 |
| Ostrya | 22 | | | | | | | | 23 | | | 4 | 49 |
| Palmae | | | | | | | | | | | | 12 | 12 |
| Pinaceae | 337 | 1467 | 123 | 44 | 23 | 33 | 24 | 36 | 4 | 12 | 2 | 1298 | 3403 |
| Platanus | 13 | | | | | | | | | | | 22 | 35 |
| Populus | 4 | 10 | | | | | | | 7 | | | 4 | 25 |
| Quercus | 712 | 194 | 9 | 4 | | | | | | | | 15 | 934 |
| Rosaceae | 39 | 123 | 4 | 4 | 4 | | | | | 4 | | 17 | 195 |
| Salix | 99 | 19 | 7 | | | | | | | | | 8 | 133 |
| Tilia | | 4 | | | | | | | | | | | 4 |
| Ulmus | | 5 | | | | | | | | | 15 | | 20 |
| Vitiaceae | 4 | 71 | | | | | | | | | | | 75 |
| Corylus | | | | | | | | | 10 | | | | 10 |

increase in January, peaking between February and May. They started to decrease after June.

### Herb and Gramineae Pollens

Gramineae family was the dominant pollen in this group. *Artemisia*, *Chenopodiaceae/Amaranthaceae*, *Compositae*, *Leguminosae*, *Plantago* and *Umbelliferae* were other pollens observed as grass pollens. Herb pollen begun to increase from March, reached to maximum levels at May and than slowly decreased after September, decreased gradually after October. As for Gramineae pollens, they were pronounced at a low to moderate level throughout the year. Gramineae pollens showed

TABLE IV The meteorological variables according to months

| Month | January | February | March | April | May | June | July | August | September | October | November | December |
|---|---|---|---|---|---|---|---|---|---|---|---|---|
| Temperature (°C) | | | | | | | | | | | | |
| Mean | 7.8 | 15.9 | 14.5 | 16.4 | 21.5 | 26.6 | 28.5 | 29.2 | 26.7 | 22.0 | 13.9 | 14.0 |
| Median | 7.5 | 12.2 | 14.0 | 16.8 | 22.7 | 26.6 | 28.8 | 29.4 | 26.5 | 23.5 | 16.5 | 11.0 |
| SD | 2.19 | 18.9 | 2.34 | 2.14 | 4.32 | 1.57 | 1.06 | 1.05 | 1.08 | 3.56 | 4.30 | 18.1 |
| Minimum | 3.90 | 8.40 | 10.20 | 11.5 | 10.9 | 23.5 | 26.0 | 25.7 | 25.0 | 15.0 | 6.5 | 4.2 |
| Maximum | 12.50 | ** | 17.9 | 20.0 | 27.4 | 29.4 | 30 | 31 | 28.9 | 27.8 | 20.5 | ** |
| Humidity (%) | | | | | | | | | | | | |
| Mean | 66.11 | 64.6 | 67.7 | 75.2 | 60.0 | 61.7 | 75.2 | 75.2 | 71.4 | 58.5 | 67.4 | 78.0 |
| Median | 68.3 | 66.0 | 70.7 | 75.7 | 59.0 | 63.3 | 76.7 | 75.7 | 72.1 | 63.3 | 71.6 | 80.3 |
| SD | 15.9 | 16.5 | 13.0 | 8.99 | 18.1 | 12.5 | 5.2 | 3.31 | 4.77 | 13.8 | 14.5 | 12.5 |
| Minimum | 35.3 | 35.7 | 31.3 | 50.7 | 20.7 | 35.7 | 60.3 | 68.0 | 57.3 | 26 | 33.2 | 42.7 |
| Maximum | 95.5 | 97.3 | 87.7 | 88.0 | 94.7 | 77.3 | 82.7 | 82.0 | 77.7 | 74 | 92.3 | 94.7 |
| Rain (mm) | | | | | | | | | | | | |
| Mean | 3.51 | 2.19 | 1.38 | 2.96 | 4.52 | | | 1.03 | 1.10 | 0.44 | 2.80 | 10.56 |
| SD | 8.42 | 6.15 | 3.24 | 6.04 | 9.13 | | | 4.021 | 4.06 | 0.96 | 7.17 | 15.00 |
| Minimum | 0.00 | 0.00 | 0.00 | 0.00 | 0.00 | | | 0.00 | 0.00 | 0.00 | 0.00 | 0.00 |
| Maximum | 33.0 | 28.50 | 14.10 | 30.10 | 33.2 | | | 21.20 | 18.10 | 10.60 | 28.50 | 52.40 |
| Wind (m/s) | | | | | | | | | | | | |
| Mean | 1.01 | 1.16 | 1.25 | 1.20 | 1.39 | 1.49 | 1.44 | 1.22 | 1.11 | 1.01 | 1.23 | 1.19 |
| Median | 0.70 | 0.95 | 1.20 | 1.10 | 1.00 | 1.45 | 1.40 | 1.20 | 1.10 | 0.90 | 0.85 | 0.80 |
| SD | 1.11 | 0.86 | 0.74 | 0.43 | 0.92 | 0.46 | 0.38 | 0.30 | 0.38 | 0.57 | 1.02 | 0.90 |
| Minimum | 0.00 | 0.00 | 0.10 | 0.50 | 0.30 | 0.80 | 0.90 | 0.70 | 0.40 | 0.20 | 0.10 | 0.00 |
| Maximum | 52.40 | 2.90 | 3.30 | 2.40 | 5.00 | 2.90 | 2.30 | 1.80 | 2.10 | 2.50 | 4.70 | 2.80 |

a peak in May. Similarly, Gramineae pollens started to decrease in October.

Adana is a typical Mediterranean city. Meteorologic variability is also similar to the other Mediterranean country. Meteorological levels monthly and seasonal are given below. (Tables IV and V)

In general, meteorological variability does not affect the pollens counts. As regards to pollen types, Gramineae pollen in March and May and herb pollen in January, April, May and July were associated with temperature and humidity at a statistically significant level ($R = 0.250$, $p < 0.05$). It was interesting to observe a significant increase in pollen counts on days with high humidity, following rainy days in hot seasons. The most common tree pollens were *Pineaceae*, *Cupressaceae/Taxaceae* and *Eucalyptus* in this survey. The relation between these tree pollens and meteorological variability were given in Table VI. A positive correlation was shown between these three tree pollens and two variables (temperature and humidity) in April and May ($R = 0.250$, $p < 0.05$). The most common herb pollen was *Chenopodiaceae/Amaranthaceae* family. But this pollen was not significantly correlated with meteorological variables.

The distribution of pollen according to months and relations to meteorological variably were given in Table VII and Figs. 2–5, respectively.

We found positive correlation between pollen count and symptom scores. The correlation between different pollens and symptom scores are given in Table VIII.

The relation of pollen counts (total, Gramineae, Herb and tree pollen) and, meteorological variability and symptom scores are shown in Figs. 2–5.

Positive correlations were seen between both Gramineae and Herb pollens, and humidity and rainfall from March to July. However, for tree pollens positive correlations were detected between pollen count and temperature and humidity in May and June.

### DISCUSSION

Analyses of aeroallergens have been made since 1873 (Dvorin *et al.*, 2001). Mediterranean country is a geographically complex continent with a widely differing climate and a wide spectrum of vegetation. While some plants were thought to be characteristic of a particular region, clinical and aerobiological studies have clearly

TABLE V The mean temperature and humidity according to season in Adana

| | | Temperature °C | | | Humidity % | | |
|---|---|---|---|---|---|---|---|
| | Rainless day/season | Min | Max | Mean ± SD | Min | Max | Mean ± SD |
| Winter | 56 | 3.9 | 16.7 | 10.25 ± 2.75 | 35.3 | 97.3 | 69.81 ± 16.28 |
| Spring | 50 | 10.2 | 27.4 | 17.49 ± 4.28 | 20.7 | 94.7 | 67.6 ± 16.28 |
| Summer | 92 | 23.5 | 31.0 | 28.12 ± 1.65 | 35.7 | 82.0 | 70.79 ± 9.98 |
| Autumn | 77 | 6.5 | 28.9 | 20.77 ± 6.20 | 10.2 | 92.3 | 64.51 ± 13.17 |

TABLE VI The correlation between prominent pollens and meteorological variables

| Month | Cupresseae | | | | Pineaceae | | | | Eucalyptus | | | | Chenopodiaea | | | |
|---|---|---|---|---|---|---|---|---|---|---|---|---|---|---|---|---|
| | Temperature | Humidity | Rain | Wind | Temperature | Humidity | Rain | Wind | Temperature | Humidity | Rain | Wind | I | N | Y | |
| January | ,324,060 | −,364,044 | −,260,158 | 106570 | | | | | | | | | | | | |
| February | ,149,449 | ,265,174 | ,194,322 | −,052,794 | | | | | | | | | | | | |
| March | −,098,599 | −,214,248 | ,162,375 | ,164,471 | −,239,195 | ,018,922 | −,248,178 | ,309,091 | | | | | | | | |
| April | ,530,003 | −,167,377 | −,185,328 | −,054,777 | ,480,007 | −,319,086 | −,258,168 | −,004,983 | −,458,011 | −,160,400 | −,167,379 | −,034,857 | | | | |
| May | ,149,423 | −,372,039 | −,167,370 | −,053,779 | ,402,025 | −,326,073 | −,174,348 | −,006,975 | ,128,493 | −,397,027 | −,116,535 | ,073,698 | | | | |
| June | ,114,547 | ,014,940 | , | ,157,407 | ,263,161 | −,043,822 | , | ,179,343 | ,266,156 | −,197,298 | , | −,254,176 | | | | |
| July | | | | | | | | | | | | | | | | |
| August | | | | | | | | | | | | | −,302,099 | −,507,004 | ,075,694 | −,205,269 |
| September | | | | | | | | | | | | | −,172,364 | −,353,056 | ,011,955 | −,024,900 |
| October | | | | | | | | | | | | | | | | |
| November | | | | | | | | | | | | | | | | |
| December | | | | | | | | | | | | | | | | |

TABLE VII The distribution of pollen groups according to months and relations to meteorological variably (*R* level at first line and *p* level at second line were given)

| Month | Gramineae | | | | Herbs | | | | Tree | | | |
|---|---|---|---|---|---|---|---|---|---|---|---|---|
| | Temperature | Humidity | Rain | Wind | Temperature | Humidity | Rain | Wind | Temperature | Humidity | Rain | Wind |
| January | ,225,224 | −,020,913 | −,089,635 | 0,0001 | −,028,882 | ,039,834 | ,390,030 | −,032,864 | ,216,243 | −,542,002 | −,137,461 | ,325,074 |
| February | −,155,431 | −,274,158 | −,089,652 | ,119,545 | −,012,952 | −,155,431 | ,399,078 | ,012,952 | ,149,449 | ,265,174 | ,194,322 | −,052,794 |
| March | −,461,009 | ,170,361 | −,428,016 | ,314,086 | −,393,029 | ,041,829 | −,241,192 | ,429,009 | −,260,158 | −,027,886 | −,062,741 | ,285,120 |
| April | ,282,131 | −,088,643 | −,113,551 | ,097,610 | ,409,025 | −,193,307 | −,129,498 | ,015,939 | ,491,006 | −,269,151 | −,198,294 | ,041,831 |
| May | ,491,005 | −,556,001 | −,259,160 | ,052,780 | ,314,086 | −,539,002 | −,269,144 | ,066,725 | ,258,161 | −,478,007 | −,219,236 | ,044,814 |
| June | ,332,073 | −,031,871 | – | ,064,737 | ,349,059 | −,047,807 | – | −,111,559 | ,301,106 | −,115,546 | | |
| July | −,647,000 | −,018,923 | – | ,414,021 | −,298,103 | −,132,478 | – | ,071,706 | −,536,002 | ,030,873 | | ,578,001 |
| August | ,124,508 | ,127,496 | −,057,764 | −,130,485 | −,286,118 | −,368,042 | ,090,638 | −,181,330 | ,562,001 | ,504,004 | −,172,364 | −,025,894 |
| September | −,290,120 | −,268,153 | −,134481 | −,061,747 | ,005,977 | ,016,933 | ,095,619 | ,192,310 | −,101,595 | −,049,797 | −,214,256 | −,238,205 |
| October | ,046,807 | ,008,967 | ,775,225 | ,220,234 | −,208,261 | ,019,920 | ,775,225 | ,162,384 | −,241,191 | ,215,246 | ,632,368 | −,040,832 |
| November | −,186,324 | −,202,285 | −,122,512 | ,128,493 | −,017,929 | −,125,512 | −,242,191 | −,017,929 | −,213,259 | ,088,645 | ,016,933 | −,006,974 |
| December | ,051,784 | ,025,892 | ,018,923 | ,196,291 | ,112,547 | −,060,747 | ,211,255 | ,290,113 | ,222,230 | ,104,579 | ,453,011 | ,318,081 |
| I* | −,543,266 | −,754,084 | ,551,257 | ,143,787 | −,257,623 | −,261,618 | ,667,148 | −,371,468 | −,657,156 | −,812,050 | ,667,148 | |
| II** | ,696,125 | ,174,742 | −,406,425 | −,279,592 | ,486,329 | ,029,957 | −,371,468 | −,551,257 | −,543,266 | −,029,957 | −,143,787 | |

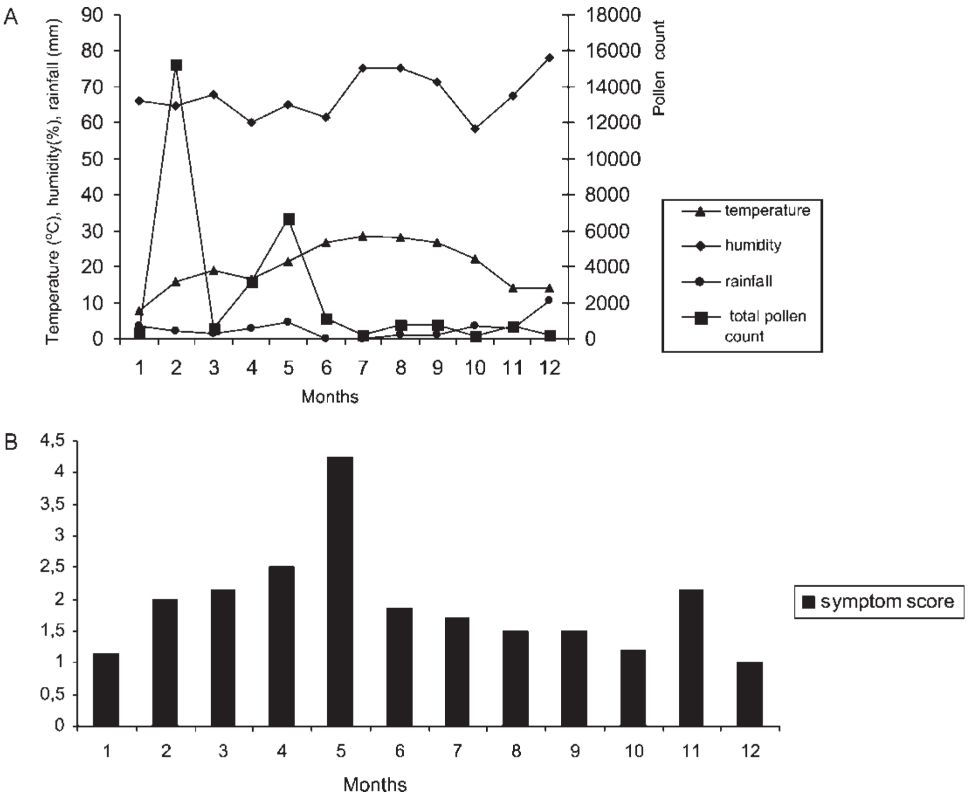

FIGURE 2 (A) The changing of pollen counts with temperature (°C), humidity (%) and rain (mm$^3$). (B) Symptom scores according to months.

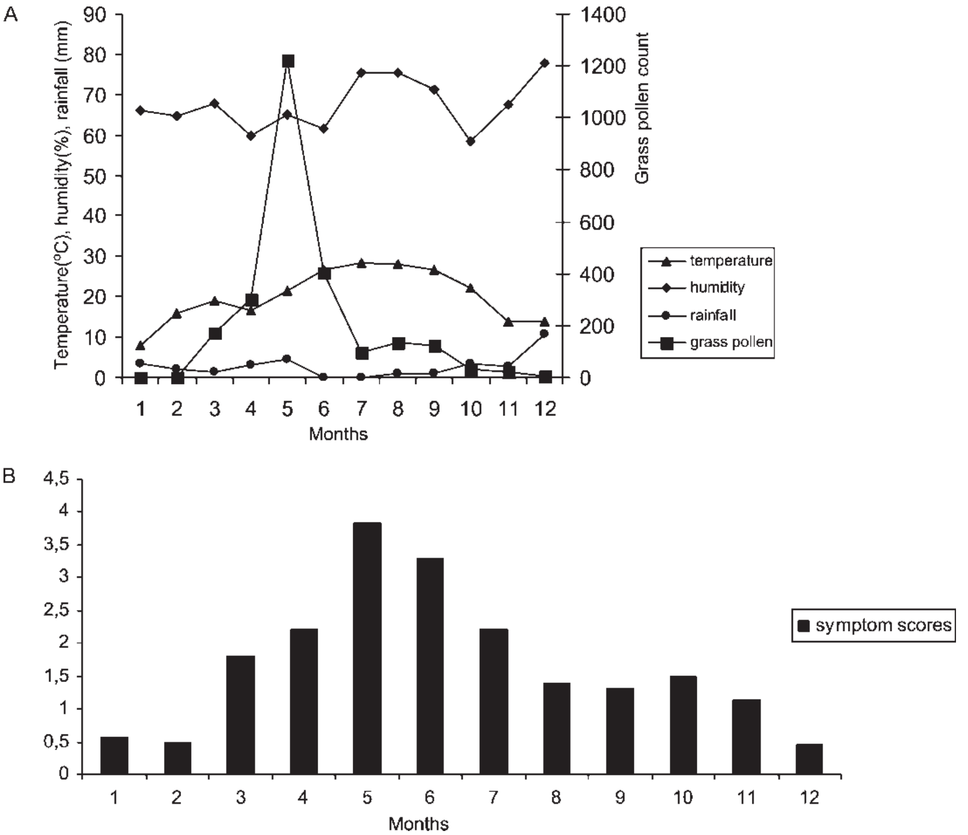

FIGURE 3 (A) The changing of grass pollen counts with temperature (°C), humidity (%) and rain (mm$^3$). (B) Symptom scores of grass pollen allergic children according to months.

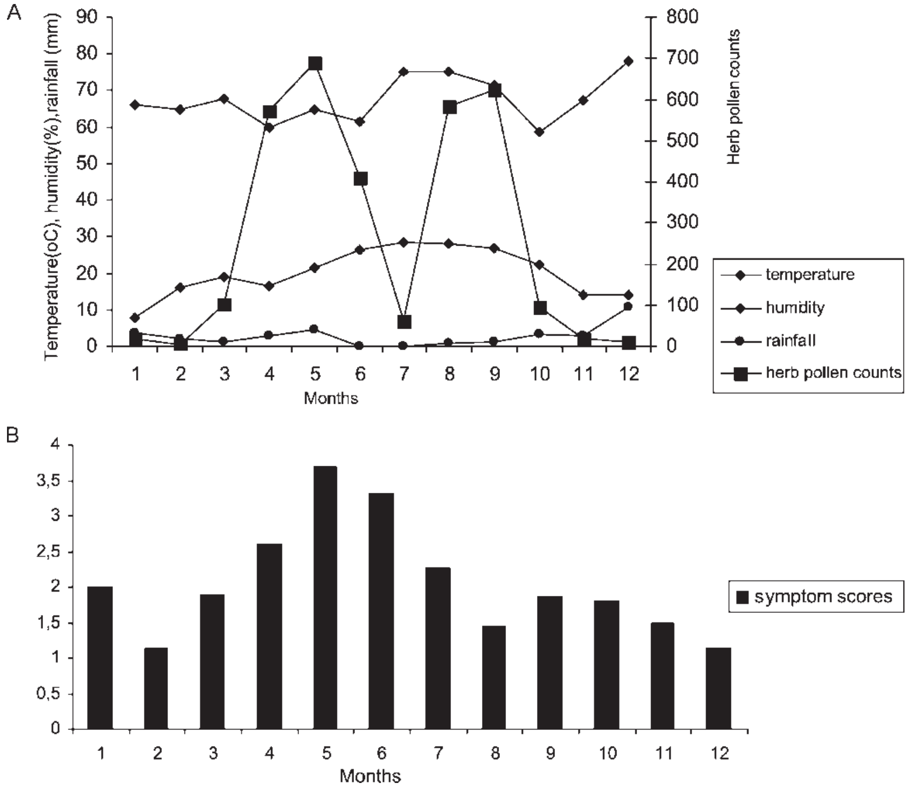

FIGURE 4 (A) The changing of herb pollen counts with temperature (°C), humidity (%) and rain (mm$^3$). (B) Symptom scores of herb pollen allergic children according to months.

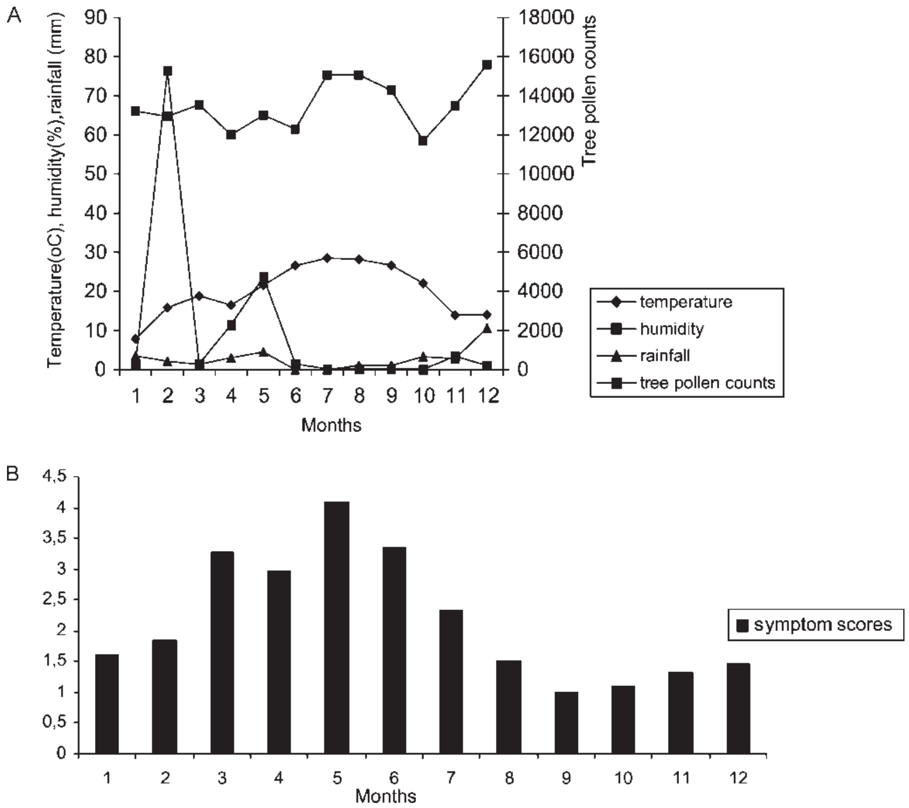

FIGURE 5 (A) The changing of herb pollen counts with temperature (°C), humidity (%) and rain (mm$^3$). (B) Symptom scores of tree pollen allergic children according to months.

TABLE VIII The relationship between pollen concentration and SS

| S S × grains/m$^3$ | *R* | *P* |
|---|---|---|
| SS of tree allergic children × tree pollen counts | 0.585 | 0.046 |
| SS of Grass allergic children × Grass pollen counts | 0.655 | 0.021 |
| SS of Gramineae allergic children × Gramineae pollen counts | 0.872 | 0.000 |
| SS of Eucalyptus allergic children × Eucalyptus pollen counts | 0.899 | 0.000 |
| SS total × total pollen counts | 0.646 | 0.023 |

shown that the pollen maps of Europe and Mediterranean are changing, partly because of the international trade in plants for gardening purposes.

With a considerable length of coasts in the Mediterranean region, Turkey is a country, which connects Asia and Europe. Pollen in atmosphere was first counted in 1967 in Ankara (Ozkaragöz, 1967), which is located in the centre of Anatolia. Following this survey, since 1994 (İnceoğlu *et al.*, 1994), most common pollens have been studied in Turkey, which has various geographic characteristics (Ozkaragöz, 1967; İnceoğlu *et al.*, 1994; Pınar *et al.*, 1999; Guvensen and Ozturk, 2003). This study is the first one done in Adana, which is a typical Mediterranean city with 14,030 m$^2$ surface area. In addition to the typical Mediterranean vegetation, the city is surrounded by the Taurus covered with Pineacea. It has been reported that pollens, following mites, are the second most frequent air-borne allergens (Güneser *et al.*, 1996). Although the typical vegetation in region has long been known, there is no study in Adana.

When pollen map of our area is compared to the other European pollen maps, *Cupressaceae* pollens were shown for a longer period from November to the end of June than those in Europe. *Betula* pollen was observed for a shorter period than the ones in Middle European Countries in November and December. Between March and June, *Oleaceae* pollen distribution is similar to those in other European Countries. Gramineae species were shown throughout year. *Urticaceae* pollen were shown between March and June, which is a period shorter compared to the ones in other European countries. *Compositae* pollen was found in different months from that of Europe. (March to November in Turkey and July to December in Europe). *Chenopodiaceae/Amaranthaceae* family was also shown for a very long period in Adana.

Similar types of pollens were identified in our study when compared with the Mediterranean region. The difference in their pollen concentrations is not significant. In general, tree pollen with a percentage of 82% is slightly higher than that of the inner (76%) and other parts of Turkey. Also, tree pollen was dominant in the Ankara atmosphere forming 76% of the total amount of pollen collected over the period 1990–1993 with Gramineae 14%, and herb 10% (İnceoğlu *et al.*, 1994). However the pollinisation period was longer in our region. In addition, pollinisation period of Parietaria was found to be shorter, and this period for *Compasitae* was longer which was different than other Mediterranean countries.

Fluctuation in pollen levels were not significantly correlated with any meteorological variable examined in this study. Similarly, literature also determined that tree pollen was seemingly unaffected by changes in temperature. However, in different studies positive correlation was found between pollen count and warm temperature and precipitation (Epton *et al.*, 1997; Çelik *et al.*, 2003; Sin *et al.*, 2003). This is a preliminary study and effects of meteorological variables on pollen counts could not be clearly identified with the evaluation of one-year data yet. As indicated by previous researcher's examination, multiple and accumulated weather parameters for a longer period is necessary for a complete understanding of relationship between meteorology and aerobiology.

We found positive correlation between pollen counts and symptom scores in pollen allergic children (Table VII). Our patient group included only pollen sensitive children and so, this correlation is not surprising. The previous studies had conflicting data with effects of pollen counts on symptom scores and admission to hospital or emergency departments (Epton *et al.*, 1997; Çelik *et al.*, 2003; Sin *et al.*, 2003). In these surveys, the atopic origin of patients was not clear and non atopic patients with asthma were enrolled to the study. In addition, only asthmatic patients had been studied. In their study, Epton *et al.* found small effects of aeroallergens on PEFR and asthma symptoms and they thought that other causes need to be sought to account for variations in asthma severity and exacerbations (Epton *et al.*, 1997). Allergic rhinitis is another important allergic disease and has been frequently come across entity at least asthma in allergy clinics. Especially, pollen sensitivity related rhinitis and rhinoconjunktivitis and asthma is responsible for high morbidity in children and adolescent. Our study group consisted of only pollen allergic children with asthma and/or allergic rhinitis. For this reason, we thought that it may help to understand the real effects of pollen counts on allergic diseases. Other factors responsible for fluctuation of allergic disease might be excluded by this way.

We suggest that further studies in longer periods and in larger populations should be done to determine the relation between aerobiology and meteorological variables and allergic diseases.

### CONCLUSION

In our region, pollen map and its changes in accordance with the climatic changes have not been studied before.

In this study, we investigated the types of pollen and their changes according to the months and meteorological data.

Adana, which has all the characteristics of Mediterranean region has shown high pollen counts, which is the vegetation specific to this region. To have knowledge about distribution of pollen concentration according to months is especially important for our city where the prevalence of asthma and allergic rhinitis is 12.7 and 13.6%, respectively. In this one-year study, it was observed that there was a significant increase in pollen counts on days with high humidity, following rainy days in hot seasons. We have planned to continue this survey to predict pollen concentration in future.

### Acknowledgements

This study was supported by TÜBİTAK (SBAG2345).

### References

Bousquet, J., Guerin, B., Dottle, A., *et al.* (1985) "Comparison between rush immunotherapy with standardized allergen and an alum adjuved pyridine extracted material in grass pollen allergy", *Clin. Allergy* **15**, 191–193.

Çelik, G., Mungan, D., Abadoğlu, O., Pınar, N.M. and Mısırlıgil, Z. (2003) Direct medical cost assasment in subjects with seasonal allergic rhinitis living in Ankara, Turkey. Allergy Asthma Proceeding (in press)

D'amato, Spieksma, F. Th. M. and Bonini, S. (1991) Allergenic pollen and pollinosis in Europea (Blackwell Science, Oxford, UK), pp 63–65.

D'amato, G., Spieksma, F.T.H.M., Liccardi, G. *et al.* (1998) Pollen-related allergy in Europe. Position Paper of the European Academy of Allergology and Clinical Immunology Allergy 53, 567–578.

Dvorin, D.J., Lee, J.J., Belecanech, G.A., *et al.* (2001) "A comparative, volumetric survey of airborne pollen in Philedelphia, Pennsylvania (1991–1997) and Cherry Hill, New Jersey (1995–1997)", *Annals Asthma Allergy Immunol.* **87**, 394–404.

Epton, M.J., Martin, I.R., Graham, P., *et al.* (1997) "Climate and aeroallergen levels in asthma: a 12-month prospective study", *Thorax* **52**, 528–534.

ERCHS (1996) "Variations in the prevalence of respiratory symptoms, self reported asthma attacks and use of asthma medications in European Community Respiratory Healthy Survey", *Eur. Respir. J.* **9**, 687–695.

Güneşer, S., Atıcı, A., Cengizler, I. and Alparslan, N. (1996) "Inhalant allergens: as a cause of respiratory allergy in east Mediterranean area Turkey", *Allergol. Immonopathol.* **24**(3), 116–119.

Guvensen, A. and Ozturk, M. (2003) "Airborne pollen calendar of İzmir-Turkey", *Ann. Agric. Environ. Med.* **10**(1), 31–36.

İnceoğlu, Ö., Pınar, N.M., Şakıyan, N. and Sorkun, K. (1994) "Airborne pollen concentration in Ankara, Turkey 1990–1993", *Grana* **33**, 158–161.

ISAAC. Steering committee (1998) "Worldwide variation in prevalence of symptoms of asthma, allergic rhino conjunctivitis and atopic eczema", *Lancet* **351**, 1225–1232.

Ozkaragöz, K. (1967) "Pollens, mold spores, and other inhalants as etiologic agents of respiratory allergy in the central part of Turkey", *J. Allergy* **40**(1), 21–25.

Pınar, N.M., Sakıyan, N., İnceoğlu, Ö. and Kaplan, A. (1999) "A one-year aeropalynological study at Ankara, Turkey", *Aerobiologia* **15**, 307–310.

Sin, B., Çelik, G., Pınar, N.M., Mungan, D. and Mısırlıgil, Z. (2003) "Is preseasonal immunotherapy effective in patients with respiratory allergy?", *EAACI* **57**(73), 53.

